# MASHt: Software for statistical analysis of transcriptomes' qualitative features in factorial experiments

**DOI:** 10.6026/973206300213162

**Published:** 2025-09-30

**Authors:** Maria Nuc, Michal Stanoch, Hanna Cwiek-Kupczynska, Barbara Naganowska, Pawel Krajewski

**Affiliations:** 1 Institute of Plant Genetics, Polish Academy of Sciences, Strzeszynska 34, 60-479 Poznan, Poland; 2 Luxembourg Centre for Systems Biomedicine, University du Luxembourg, 1 Bd du Jazz, 4370 Esch-Belval, Luxembourg

**Keywords:** Transcriptomics, factorial experiments, sequence variation, software tools

## Abstract

In Next-Generation Sequencing (NGS) data analysis, attention is given more often to the variation in gene expression levels in several experimental variants than to the qualitative differences between transcriptomes. We present the MASHt toolkit for analyzing qualitative variation in sequencing data from multifactorial experiments. The analysis involves computing pairwise Mash distances between datasets, conducting principal coordinate analysis of the resulting distance matrix, and applying univariate and multivariate analyses of variance to the principal coordinates. MASHt supports computations on data subsets, such as those annotated by a specific ontology. We demonstrate the use of MASHt with an example experiment examining the impact of temperature on the transcriptomes of different barley genotypes.

## Background:

Transcriptomics most often focuses on differences in NGS-derived transcript quantities between samples representing various genotypes or conditions, and numerous tools exist for such differential gene expression analysis [1]. However, transcriptomic data are less frequently used to identify qualitative differences between sequences characteristic of different organisms, cell types, or conditions, which encompass the variability in the presence/absence patterns of transcripts, alternative splicing, or structural variations; these may arise from genomic variations, genetic mosaicism or chimerism, chromatin structure differences, or post-transcriptional modifications [2]. Additionally, biotic and abiotic environmental factors may affect transcript maturation mechanisms [3]. In simple case/control designs, finding specific polymorphisms may be sufficient for the inference, while in experiments involving many (possibly interacting) factors, the analysis should be based on statistical models appropriate for studying the variation between treatments relative to the background variation, considering the experimental design [4]. The proposed MASHt toolkit identifies significant qualitative differences in sequencing data from multifactorial experiments using linear models; it utilizes Mash distances [5], which measure qualitative differences by identifying common and unique k-mers between datasets. Then, it conducts principal coordinate analysis (PCoA; [6]) on the resulting distance matrix to transform qualitative differences into quantitative variables and applies univariate and multivariate analyses of variance to the principal coordinates. MASHt supports the analysis of datasets annotated with a chosen ontology, facilitating functional interpretation of qualitative differences [7]. Therefore, it is of interest to describe the design and development of software for statistical analysis of transcriptomes' qualitative features in factorial experiments.

## Program input:

The input consists of sets of nucleotide sequences. The package includes three modules-mash, stats, blaster-each automating a specific step of the analysis.

## Mash:

Compute Mash distances. Many types and formats of input data are allowed, including fasta and fastq, containing long (*e.g.*, scaffolds or full-length transcripts) or short (*e.g.*, sets of NGS reads) sequences.

## Stats:

It performs PCoA to represent the data structure in a low-dimensional space on the basis of calculated distances. The subsequent ANOVA/MANOVA tests, based on an input file containing the description of the experimental design and linear model formula, assess whether experimental factors or their interactions account for the observed variability.

## Blaster:

Splits observed sequences into groups based on their mapping to a reference set. The inputs for this module include a file containing reference sequences annotated with features of interest (*e.g.*, GO or GOSlim terms).

## Program output:

To illustrate the MASHt approach and output, we used RNA-seq data obtained in the experiment performed to examine the impact of heat stress on the barley transcriptome [8]. Three genotypes were investigated ('Bowman' cultivar, BW, and its two near-isogenic lines, BW827 and BW828) under two treatments: optimal temperature (8°C/16°C night/day from sowing to the end of tillering) and high temperature (28°C night/day in the same period). Samples were collected at two timepoints-the start of the tillering stage and ten days later-in three biological replicates. The data were assembled into scaffolds using SOAPdenovo2 [9]. The primary outputs obtained from MASHt were the distance matrix between all pairs of sets of scaffolds, PCoA results with low-dimensional plots (Figure 1A - see PDF) and ANOVA/MANOVA results (Table 1 - see PDF). The analysis showed that day, temperature, and their interaction had statistically significant effects on the distribution of the data points (Figure 1A - see PDF). In general, points associated with the optimal temperature have lower values of PC1 than those associated with high temperature, and points associated with the first day of tillering have lower values of PC2 than those associated with the tenth day. To perform the part of the analysis related to functional annotation, the reference set of barley transcript isoforms, annotated with GOSlim terms, was downloaded from the Ensembl Plants database. Each of the target datasets was divided into subsets containing scaffolds linked to a particular term, and the analysis was performed for these subsets (with Bonferroni correction relative to the number of terms). The results of this analysis are presented (Figure 1B - see PDF) in the GOSlim ontology graph.

## Caveat:

MASHt is implemented as a Python package; it uses the MASH executable [5] for computing distances and BLAST+ [10] for grouping the observations on the basis of annotation according to ontology terms. For good inference, experimental designs involving biological replicates are recommended. The results of the analysis-significant changes in nucleotide sequences between various experimental variants-can be interpreted by themselves to infer biological conclusions from the experiments or as additional explanatory information in other analyses, *e.g.*, in quantitative differential expression analysis or in genetic variant analysis. Computations in MASHt are fast, so its availability should help with the incorporation of qualitative methods into sequence analysis. Although developed for RNA-seq data in multifactorial experiments, MASHt is also applicable to DNA-seq data with appropriate interpretative adjustments.
